# Batten disease: biochemical and molecular characterization revealing novel *PPT1* and *TPP1* gene mutations in Indian patients

**DOI:** 10.1186/s12883-018-1206-1

**Published:** 2018-12-12

**Authors:** Jayesh Sheth, Mehul Mistri, Riddhi Bhavsar, Dhairya Pancholi, Mahesh Kamate, Neerja Gupta, Madhulika Kabra, Sanjiv Mehta, Sheela Nampoothiri, Arpita Thakker, Vivek Jain, Raju Shah, Frenny Sheth

**Affiliations:** 1 FRIGE’s Institute of Human Genetics, FRIGE House, Jodhpur Gam Road, Satellite, Ahmedabad, Gujarat 380015 India; 2 Department of Pediatric Neurology, KLES Prabhakar Kore Hospital, Belgaum, Karnataka 590010 India; 3 Division of Genetics (Pediatrics), All India Institute of Medical Sciences, New Delhi, 110029 India; 4 Usha-Deep Children Neurology and Epilepsy clinic, Ahmedabad, 380014 India; 5 Department of Pediatric Genetics, Amrita Institute of Medical Science and Research Centre, Kochi, Kerala 682041 India; 6 Department of Neurology, Lokmanya Tilak Medical College, Sion Hospital, Mumbai, Maharashtra 400022 India; 7 Department of Neurology, Santokba Durlabhji Hospital, Jaipur, 302015 Rajasthan India; 8 Ankur Neonatal Nursery, Ahmedabad, 380009 Gujarat India

**Keywords:** PPT1, TPP1, Batten disease, Neuronal ceroid lipofuscinoses (NCL)

## Abstract

**Background:**

Neuronal ceroid lipofuscinoses type I and type II (NCL1 and NCL2) also known as Batten disease are the commonly observed neurodegenerative lysosomal storage disorder caused by mutations in the *PPT1 and TPP1* genes respectively. Till date, nearly 76 mutations in *PPT1* and approximately 140 mutations, including large deletion/duplications, in *TPP1* genes have been reported in the literature. The present study includes 34 unrelated Indian patients (12 females and 22 males) having epilepsy, visual impairment, cerebral atrophy, and cerebellar atrophy.

**Methods:**

The biochemical investigation involved measuring the palmitoyl protein thioesterase 1 and tripeptidy peptidase l enzyme activity from the leukocytes. Based on the biochemical analysis all patients were screened for variations in either *PPT1* gene or *TPP1* gene using bidirectional Sanger sequencing. In cases where Sanger sequencing results was uninformative Multiplex Ligation-dependent Probe Amplification technique was employed. The online tools performed the protein homology modeling and orthologous conservation of the novel variants.

**Results:**

Out of 34 patients analyzed, the biochemical assay confirmed 12 patients with NCL1 and 22 patients with NCL2. Molecular analysis of *PPT1* gene in NCL1 patients revealed three known mutations (p.Val181Met, p.Asn110Ser, and p.Trp186Ter) and four novel variants (p.Glu178Asnfs*13, p.Pro238Leu, p.Cys45Arg, and p.Val236Gly). In the case of NCL2 patients, the *TPP1* gene analysis identified seven known mutations and eight novel variants. Overall these 15 variants comprised seven missense variants (p.Met345Leu, p.Arg339Trp, p.Arg339Gln, p.Arg206Cys, p.Asn286Ser, p.Arg152Ser, p.Tyr459Ser), four frameshift variants (p.Ser62Argfs*19, p.Ser153Profs*19, p.Phe230Serfs*28, p.Ile484Aspfs*7), three nonsense variants (p.Phe516*, p.Arg208*, p.Tyr157*) and one intronic variant (g.2023_2024insT). No large deletion/duplication was identified in three NCL1 patients where Sanger sequencing study was normal.

**Conclusion:**

The given study reports 34 patients with Batten disease. In addition, the study contributes four novel variants to the spectrum of *PPT1* gene mutations and eight novel variants to the *TPP1* gene mutation data. The novel pathogenic variant p.Pro238Leu occurred most commonly in the NCL1 cohort while the occurrence of a known pathogenic mutation p.Arg206Cys dominated in the NCL2 cohort. This study provides an insight into the molecular pathology of NCL1 and NCL2 disease for Indian origin patients.

**Electronic supplementary material:**

The online version of this article (10.1186/s12883-018-1206-1) contains supplementary material, which is available to authorized users.

## Background

The neuronal ceroid lipofuscinoses (NCLs) are a group of inherited lysosomal storage disorder causing severe neurodegeneration due to neuronal loss (brain and retina), and accumulation of lipopigments in many cell types, including neurons. The NCLs incidence rate worldwide is 1 to 8 in 100,000 live births [1].

The NCLs share common clinical presentations like epilepsy, loss of motor and cognitive function, visual impairment, and premature death [2]. Though usually observed in childhood, the age of onset of the disease varies. Considering this, NCLs were initially classified into four groups – infantile (INCL-Haltia-Santavuori disease), late infantile (LINCL-Jansky-Bielschowsky disease), juvenile (Batten-Spielmeyer-Vogt disease) and adult (Kufs disease) [3]. Eventually, allelic heterogeneity in NCLs was identified due to an advancement in biochemical and genetic techniques and hence a new approach of molecular classification and diagnostic algorithms was designed [4, 5].

Until now, 14 types of NCL are identified (NCL1-NCL14) however the most commonly observed form are NCL1, NCL2, and NCL3 [6]. These NCLs subtypes are autosomal recessively inherited except for NCL4B, inherited in an autosomal dominant form [7]. An exception to the above inheritance pattern is a uniparental disomy case in NCL which occurred due to complete isodisomy of chromosome 8, leading to homozygosity of a maternally-inherited deletion in NCL8 [8]. Until May 2015, total 515 changes in 13 human genes have been reported in NCLs [9].

NCL1 (OMIM#256730) representing the early infantile disease, results due to a mutation in *PPT1* (palmitoyl-protein thioesterase 1; OMIM*600722) gene located at 1p34.2. The gene codes for a lysosomal enzyme called palmitoyl-protein thioesterase 1 (PPT1) whose function is to remove fatty acids attached in thioester linkages to cysteine residues in the protein. The downstream effect of PPT1 deficiency involves deregulated cellular processes like vesicular trafficking, synaptic function, lipid metabolism, neural specification, and axon connectivity [10]. In addition, a study by Lyly et al. established that an alteration in cholesterol metabolism and ectopic F1-ATP synthase resulted due to PPT1 deficiency [11]. According to the NCL mutation and patients’ database 76 changes have been reported in *PPT1* gene [9]. Amongst these, the mutation p.Arg122Trp has a founder effect in Finnish population with NCL1 [12]. This mutation causes a defect in the transport of the PPT1 from the endoplasmic reticulum to lysosomes [12, 13]. The mutation p.Thr75Pro and p.Leu10Ter have a founder effect in Scotland [14].

NCL2 (OMIM#204500), representing the late infantile disease, results due to a defect in *TPP1* (tripeptidyl peptidase I; OMIM*607998) gene at the locus 11p15.4. This gene encodes the instruction for making the lysosomal enzyme called tripeptidyl peptidase 1 (TPP1). TPP1 deficiency results in accumulation of ceroid lipofuscin, an autofluorescent storage material, in cell’s lysosomes. Total 140 disease-causing mutations are in the *TPP1* gene of the patients with NCL2 [9]. The most common *TPP1* gene mutations are c.509-1G > C and p.Arg208Ter [14]. The mutation p.Gly284Val seems to be predominant in Canada suggesting a possible founder effect [14].

The genetics of NCL1 and NCL2 remain unknown in India. Hence, the aim of the present study is to identify the molecular spectrum and common molecular marker of these diseases in Indian patients. The study also aims to support the correlation between the null or reduced enzyme activity and the mutations causing disease and clinical phenotype in NCL1 and NCL2 patients.

## Methods

### Patients

The patients in the present study were the clinical cases referred by pediatric neurologist and pediatricians from collaborating centres. The study is in accordance with the tenets of the Helsinki Declaration. The Ethics committee of the Foundation for Research in Genetics and Endocrinology (FRIGE) at the Institute of Human Genetics approved the study. As per the institutional ethics committee guidelines, a written informed consent for investigation and publication of the data was obtained from the parents/guardian of the patients. The 34 unrelated patients (22 males and 12 females) presented common clinical indications like epilepsy, cerebral atrophy, and cerebellar atrophy. They were in the age range of 4 months to 9 years at the time of investigations and were referred from different geographical/ethnic background in the time from 2015 to 2017 with a clinical suspicion of Batten disease (12 patients with NCL1, and 22 patients with NCL2). Total 16 patients (47.05%) had parental consanguinity. Table 1 provides the clinical details and the demographic profile of the patients.

**Table 1:** Clinical details and demographic profile of the patients with Batten disease (NCL1 and NCL2)

	Total patients *n* = 34	NCL1 *n* = 12	NCL2 *n* = 22
Age at the time of investigation (years)	4.46 ± 2.30	3.03 ± 1.80	5.25 ± 1.93
Gender			
Male	22 (64.7%)	8 (66.6%)	14 (63.6%)
Female	12 (35.3%)	4 (33.4%)	8 (36.4%)
Regional distribution			
East India	2 (5.9%)	0	2 (9.09%)
West India	9 (26.4%)	2 (16.7%)	7 (31.81%)
North India	3 (8.8%)	1 (8.3%)	2 (9.09%)
South India	20 (58.9%)	9 (75%)	11 (50%)
Clinical presentation			
Epilepsy	31 (91.2%)	10 (83.33%)	21 (95.45%)
Visual impairment	19 (55.9%)	3 (25%)	16 (72.72%)
Cerebral atrophy	22 (64.7%)	6 (50%)	16 (72.72%)
Cerebellar atrophy	27 (79.41%)	6 (50%)	21 (95.45%)

Data are n (%) or mean ± SD.

### Biochemical investigations

Leukocyte and genomic DNA (gDNA) isolation was carried out from six milliliters of blood, drawn from each patient in ethylenediaminetetraacetic acid (EDTA) vacutainer.

### Palmitoyl protein thioesterase 1 (PPT1) enzyme activity

The patients suspected with the NCL1 disease were investigated for the lysosomal enzyme Palmitoyl protein thioesterase 1 (PPT1; EC 3.1.2.22) activity using a previously described protocol [15]. In brief, the samples were incubated with tritinX 100, β-glucosidase, dithiothreitol and the substrate 4-Methylumbelliferyl-6-sulpho-palmitoyl-β-D-glucoside at 37^0^C for 1 h. The fluorescence was measured at 4mu.

### Tripeptidy peptidase l (TPP1) enzyme activity

The NCL2 suspected patients were investigated for the Tripeptidy peptidase l (TPP1; EC 3.4.14.9) lysosomal enzyme activity using a previously described protocol [16]. In brief, the samples were mixed with EDTA/acetate/TritonX 100 in chilled condition. The substrate Ala-Ala-Phe-7-amido-4-methylcoumarin was added on limited intervals and incubated at 37^0^C for 1 h. Chloroacetate/acetate was added following the incubation and the fluorescence was measured at 460 nm.

### Molecular investigation

#### DNA extraction

DNA isolation followed the standard salting-out method and was quantified using a QIAxpert (Cat. No: 9002340) from Qiagen [17]. Sample purification was performed using The Genomic DNA Clean & Concentrator™-25 (DCC™) Kit, from Zymo Research, Irvine, California, U.S.A (Cat. No. D4064) and were stored at −20 °C until investigation.

#### Single gene sequencing (*PPT1 and TPP1* gene)

The *PPT1* and *TPP1* genes were amplified using primer sets listed in the Additional file 1. The protocol followed 37 cycles consisting of initial denaturation (94 °C; 5 min), denaturation (94 °C; 30 s), annealing (58 °C-67 °C; 45 s), elongation (72 °C; 1 min), and final elongation (72 °C; 5 min). The polymerase chain reaction (PCR) products were run on the 2.5% agarose gel and visualized under ultraviolet transilluminator.

The capillary electrophoresis technology driven fluorescent dye-labeled genetic analysis system performed the Sanger sequencing on the Applied Biosystems™ SeqStudio™ Genetic Analyzer with SeqStudio™ Data Collection Software using a previously described protocol [18].

#### Multiplex ligation-dependent probe amplification (MLPA) analysis

The procedure followed the manufacturer’s recommendations of using gDNA (100 ng) and P470-A1 NCL probe mix (MRC-Holland, Amsterdam, the Netherlands). DNA was denatured (98 °C; 5 min) and hybridized (overnight; 60 °C) with the SALSA probe mix P470. The samples were then Ligase (54 °C; 15 min) and incubated at 98 °C for 5 min to stop the reaction. The PCR amplification was carried out with the specific SALSA FAM PCR primers. Amplified products were run on the ABI 3130 Genetic Analyzer (Applied Biosystems, USA) and the MLPA peak patterns were analyzed in control and test samples to detect the copy number differences of the exons.

### In silico analysis

#### Prediction of the functional effect of the variants

The variants identified were looked up in public databases like The Human Gene Mutation Database (http://www.hgmd.cf.ac.uk), SNP database (http://www.ncbi.nlm.nih.gov/SNP/index.html) and UCL-London’s Global university database (http://www.ucl.ac.uk/ncl). The in silico tools like MutationTaster2, SIFT, FATHMM, PolyPhen2, PROVEAN, and MutationAssessor predicted the pathogenicity of the coding and non-coding DNA variants and amino acid substitution.

#### Homology modeling, structure validation and protein stability of the novel variants

The wild-type template crystallographic structures of the PPT1 (PDB ID: 1EI9) and TPP1 (PDB ID: 1EDY) were used to calculate the Root Mean Square Deviation (RMSD) of the novel mutant protein structures [19].

#### Orthologous conservation of the residues harboring the novel variant

Clastal Omega (an online multiple sequence alignment program) aligned the *PPT1* (NP_000301) and *TPP1* (NP_000382) protein sequence of *Homo sapiens* with different species to check the conservation of the residues incorporating novel variants [19].

## Results

In the present study the clinical assessment, biochemical and molecular investigation confirmed the diagnosis of 34 patients with Batten disease (12 with NCL1 and 22 with NCL2). The most common clinical indications observed in these patients were epilepsy (91.2%). The neuroimaging study including Computed Tomography (CT scan) and/or brain Magnetic Resonance Imaging (MRI) from all the patients also revealed cerebral atrophy (64.7%), and cerebellar atrophy (79.41%).

### Biochemical investigations

A significant deficiency of PPT1 and TPP1 enzyme activity was observed in the leukocytes of twelve patients with NCL1 and twenty-two patients with NCL2 respectively. The patients’ enzyme activity reduced to 0 to 2.8% compared to the control value (Tables 2 and 3).

**Table 2:** Biochemical and molecular alalysis of patients with NCL1 disease

Patient ID	Palmitoyl protein thioesterase activity^b^ (nmol/hr./mg protein)	Molecular analysis		Allele Frequency		dbSNP reference sequence	Reference
		Variant location (*PPT1* gene^c^)	Zygosity	1000 Genomes	ExAC		
P_1_	0.0	Ex6:c.541G>A/p.V181M	Hom	NR	0.0001648	rs148412181	[19]
P_2_	3.6	Ex3:c.329A>G/p.N110S	Hom	NR	0.0001813	rs142894102	[20]
P_3_	3.6	Ex6:c.558G>A/p.W186	Com Hetz	NR	0.000008237	rs386833656	[21]
		Ex5:c.532_532delG/p.E178Nfs*13		NR	NR	rs878853325	In this study
P_4_	3.1	Ex7:c.713C>T/p.P238L	Hom	NR	NR	rs878853322	In this study
P_5_^a^	0.01						
P_6_	0.72						
P_7_	1.07						
P_8_	7.3	Ex2:c.133T>C/p.C45R	Hom	NR	NR	rs878853323	In this study
P_9_	0.36	Ex7:c.707T>A/p.V236G	Hom	NR	NR	rs878853324	In this study
P_10_	5.2	Not found	–	–	–	–	–
P_11_	7.4						
P_12_	3.1						

*Abbreviations*: *Com Hetz* Compound Heterozygous, *dbSNP* The Single Nucleotide Polymorphism database, *ExAC* The Exome Aggregation Consortium, *Ex* Exon, *Hom* Homozygous, *NR* Not Reported

^a^ Parents are found carrier for the same variant

^b^Palmitoyl protein thioesterase enzyme activity normal range: 25.5–215 nmol/hr./mg protein

^c^The above variants refers to the *PPT1* gene with transcript ID ENST00000642050.1 and reference sequence number NM_000310.3

**Table 3:** Biochemical and molecular analysis of patients with NCL2 disease

Patient ID	Tripeptidyl Peptidase-I activity^b^ (nmol/hr./mg protein)	Molecular analysis		Allele Frequency		dbSNP reference sequence	Reference
		Variant location (*TPP1* gene^c^)	Zygosity	1000 Genomes	ExAC		
P_13_	10.2	Ex8:c.1033A>C/p.M345L	Hetz	0.0030	0.001796	rs141482368	[22]
P_14_	4.5	Ex8:c.1015C>T/p.R339W	Hom	NR	0.00001648	rs750428882	[23]
P_15_	4.9	Ex8:c.1016G>A/p.R339Q	Hom	NR	0.000008241	rs765380155	[2]
P_16_	11.9	Ex12:c.1546_1547delTT/p.F516*	Hom	NR	NR	–	[24]
P_17_	5.4	Ex6:c.616C>T/p.R206C	Hom	NR	0.00001649	rs28940573	[25]
P_18_*	8.2						
P_19_	4.7						
P_20_	2.9						
P_21_^a^	0.0						
P_22_^a^	9.7	Ex6:c.622C>T/p.R208*	Hom	NR	0.0002	rs119455955	[26]
P_23_	6.5	Ex7:c.857A>G/p.N286S	Com Hetz	NR	NR	rs119455958	[27]
		Ex3:c.184delT/p.S62Rfs*19		NR	NR	NR	In this study
P_24_	9.1	Ex5:c.456G>C/p.R152S	Hom	NR	NR	rs869025274	In this study
P_25_^a^	0.0	Ex11:c.1376A>C/p.Y459S	Hom	NR	NR	rs864309505	In this study
P_26_	4.6						
P_27_^a^	0.3	Ex5:c.455_488del/p.S153Pfs*19	Hom	NR	NR	NR	In this study
P_28_^a^	0.0	Ex5:c.471C>A/p.Y157*	Hom	NR	NR	rs553522118	In this study
P_29_	9.2	Ex7:c.689_689delT/p.F230Sfs*28	Com Hetz	NR	NR	NR	In this study
		Ex12:c.1449_1450insG/p.I484Dfs*7		NR	NR	NR	In this study
P_30_^a^	0.4	In4:g.2023_2024insT	Hom	NR	NR	NR	In this study
P_31_^a^	0.0						
P_32_	3.5	Not found	–	–	–	–	–
P_33_	5.6						
P_34_	8.2						

*Abbreviations*: *Com Hetz* Compound Heterozygous, *dbSNP* The Single Nucleotide Polymorphism database, *ExAC* The Exome Aggregation Consortium, *Ex* Exon, *Hom* Homozygous, *In* Intron, *NR* Not Reported

^a^Parents are found carrier for the same variant

^b^Tripeptidyl Peptidase-I enzyme activity normal range: 32.8–233.0 nmol/hr./mg protein

^c^The above variants refers to the *TPP1* gene with transcript ID ENST00000299427.6 and reference sequence number NM_000391.3

### Molecular analysis

#### Bi-directional sanger sequencing (*PPT1* gene)

The biochemical investigation confirmed 12 patients with NCL1. Sanger sequencing identified five homozygous missense variants, one frameshift variant, and one nonsense variant from total nine patients (Table 2).

#### Known mutation detected in the *PPT1* gene

In total three known variants were revealed by Sanger sequencing. Patient P_1_ and P_2_ harbor the pathogenic homozygous mutations p.Val181Met in exon 6 and p.Asn110Ser in exon 3 respectively [20, 21]. Patient P_3_ carried compound heterozygous mutation p.Trp186Ter (known pathogenic) in exon 6 and a novel variant p.E178Nfs*13 in exon 5 [22].

#### Novel variants detected in *PPT1* gene

**Fig. 1:**
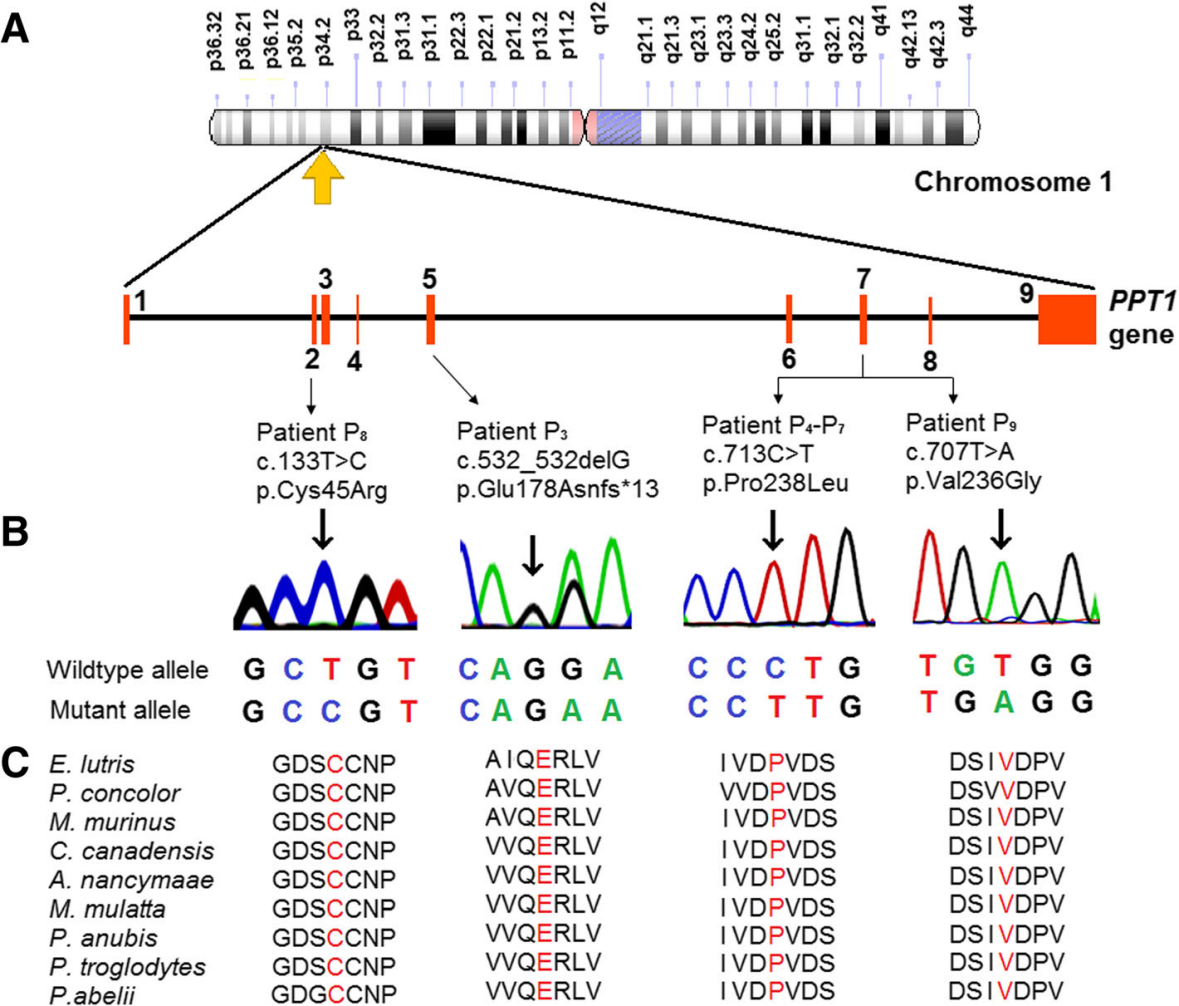
Identification of novel variants in *PPT1* gene. **a** Illustrative representation of the distributions of the novel variants identified in Indian NCL1 patients investigated in this study. **b** Sanger sequencing discovered one missense variant (p.Cys45Arg) in exon 2, two missense variants (p.Pro238Leu and p.Val236Gly) in exon 7, and one frameshift variation (p.Glu178Asnfs*13) in exon 5 of *PPT1* gene. The common variant p.Pro238Leu was identified in 44% of the patients. The point of variation is indicated by an arrow. **c** The multiple alignment of the protein sequence surrounding the novel variants against various orthologous sequence revealed the conservative status of the wildtype residues (marked red).

Overall, four novel variants amongst nine patients responsible for NCL1 were detected (Fig. 1). Patient P_4_ to P_7_ were homozygous for the variant p.Pro238Leu in exon 7. Patient P_8_ and P_9_ presented homozygous missense variants p.Cys45Arg in exon 2 and p.V236G in exon 7 respectively.

Three patients (P_10_, P_11_, and P_12_) did not carry any variation in the exon or exon-intron boundaries of *PPT1* gene but their common clinical presentations like epilepsy, cerebral atrophy, and cerebellar atrophy indicated NCL. Their biochemical analysis depicting four times decrease in the PPT1 enzyme activity confirmed the NCL1 diagnosis. Since Sanger sequencing was uninformative, these patients were analyzed through MLPA. However, no large deletion/duplication was discovered in the *PPT1* gene. In such cases, the possibility of deep intronic variations cannot be ruled out.

#### Bi-directional sanger sequencing (*TPP1* gene)

As confirmed by biochemical investigation, 22 patients affected with NCL2 were analyzed for pathogenic variants in *TPP1* gene. Overall, 15 variants, comprising seven missense variants, four frameshift variants, three nonsense variants, and one intronic variant were identified amongst 19 patients (Table 3).

#### Known mutations detected in *TPP1* gene

The *TPP1* gene analysis detected seven known mutations distributed amongst 11 patients. Patient P_13_ was identified with a heterozygous copy of the known mutation p.Met345Leu in exon 8 [23]. However, the second variant responsible for the disease was unidentified may be due to its presence in the deep intronic region or due to possible large deletion/duplication. The patient has reduced TPP1 enzyme activity and clinical phenotypes like epilepsy, regression of mental and motor milestone, choreoathetosis, cerebral atrophy, and cerebellar atrophy were in line with the diagnosis. Patient P_14_ and P_15_ carried the homozygous missense mutation p.Arg339Trp and p.Arg339Gln respectively in exon 8 [2, 24]. Patient P_16_ was detected with a nonsense mutation p.Phe516Ter in exon 12 [25]. Patient P_17_ to P_21_ harbor a homozygous missense mutation p.Arg206Cys in exon 6 [26]. Patient P_22_ suffered NCL2 due to a homozygous nonsense mutation p.Arg208Ter in exon 6 [27].

#### Novel variants detected in *TPP1* gene

**Fig. 2:**
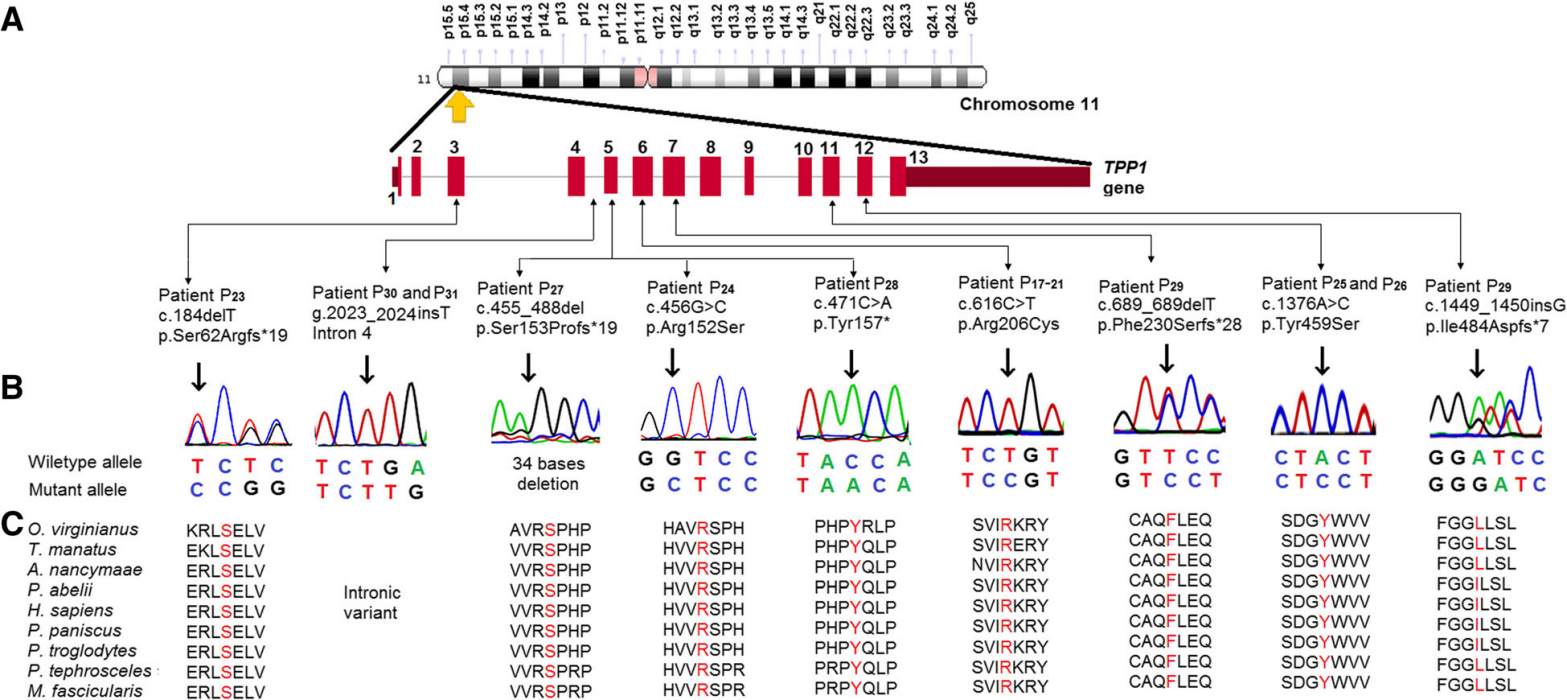
Identification of novel and most common variants in *TPP1* gene. **a** Illustrative representation of the distributions of the novel and most common variants identified in Indian NCL2 patients investigated in this study. **b** Sanger sequencing discovered one intronic variant (In4:g.2023_2024insT), three missense variants (Ex5:p.Arg152Ser, Ex11:p.Tyr459Ser and Ex6:p.Arg206Cys), four frameshift variants (Ex3:p.Ser62Argfs*19, Ex5:p.Ser153Profs*19, Ex7:p.Phe230Serfs*28, and Ex12:p.Ile484Aspfs*7), and one nonsense variant (Ex5:p.Tyr157*) in *TPP1* gene. The common variant Ex6:p.Arg206Cys occurred in 26% of the patients. The point of variation is indicated by an arrow. **c** The multiple alignment of the protein sequence surrounding the novel variants against various orthologous sequence revealed the conservative status of the wildtype residues (marked red). However, the residue isoleucine at the position 484 was found to be conserved in four out of nine species.

Sanger sequencing revealed eight novel variants amongst nine patients (patients P_23_ to P_31_). This includes four frameshift variants, two missense variants, one intronic insertion, and one nonsense variant (Fig. 2). Patient P_23_ was found with a compound heterozygous variants p.Asn286Ser (known pathogenic) and p.Ser62Argfs*19 in exon 7 and exon 3 respectively [28]. In case of the patient P_24_, a homozygous missense variant p.Arg152Ser was detected in exon 5. In the patient P_25_ and P_26,_ the variant p.Tyr459Ser in exon 11 resulted in NCL2. A frameshift termination p.Ser153Profs*19 in exon 5 was identified in the patient P_27_. Sanger sequencing detected the patient P_28_ with a homozygous nonsense termination p.Tyr157Ter in exon 5. Patient P_29_ was compound heterozygous for the variants p.F230Sfs*28 in and p.Ile484Aspfs*7 in were detected in exon 7 and exon 12 respectively. An intronic variant g.2023_2024insT intron 4 was identified in the Patient P_30_ and P_31_.

However, no variation in the exonic or exon-intronic boundaries of *TPP1* gene was detected in three patients (P_32_, P_33_, and P_34_). These patients presented common clinical indications like epilepsy, cerebral atrophy, cerebellar atrophy, and visual impairment as indicated in NCL patients. Their biochemical analysis resulting in a significant decrease in the TPP1 enzyme activity confirmed the NCL2 diagnosis. Despite the uninformative Sanger sequencing results, these patients could not be analyzed through MLPA due to unavailability of enough samples. In such cases, the possibility of deep intronic variations cannot be rule out.

### In silico analysis of the novel variants

The in silico tools described above established the functional effects of the variants identified [see Additional file 2]. The novel variants were found to be disease causing. These predicting tools suggest the probably damaging and deleterious effect of the novel variants on protein function. These variants were found neither in the 1000 Genomes database nor in the Exome Aggregation Consortium (ExAc). The protein sequence alignment of *Homo sapiens* along with other species using Clastal Omega-an online multiple sequence alignment program suggests that these variations occurred at highly evolutionarily conserved and functionally active residual domain in the protein (Figs. 1 and 2).

**Fig. 3:**
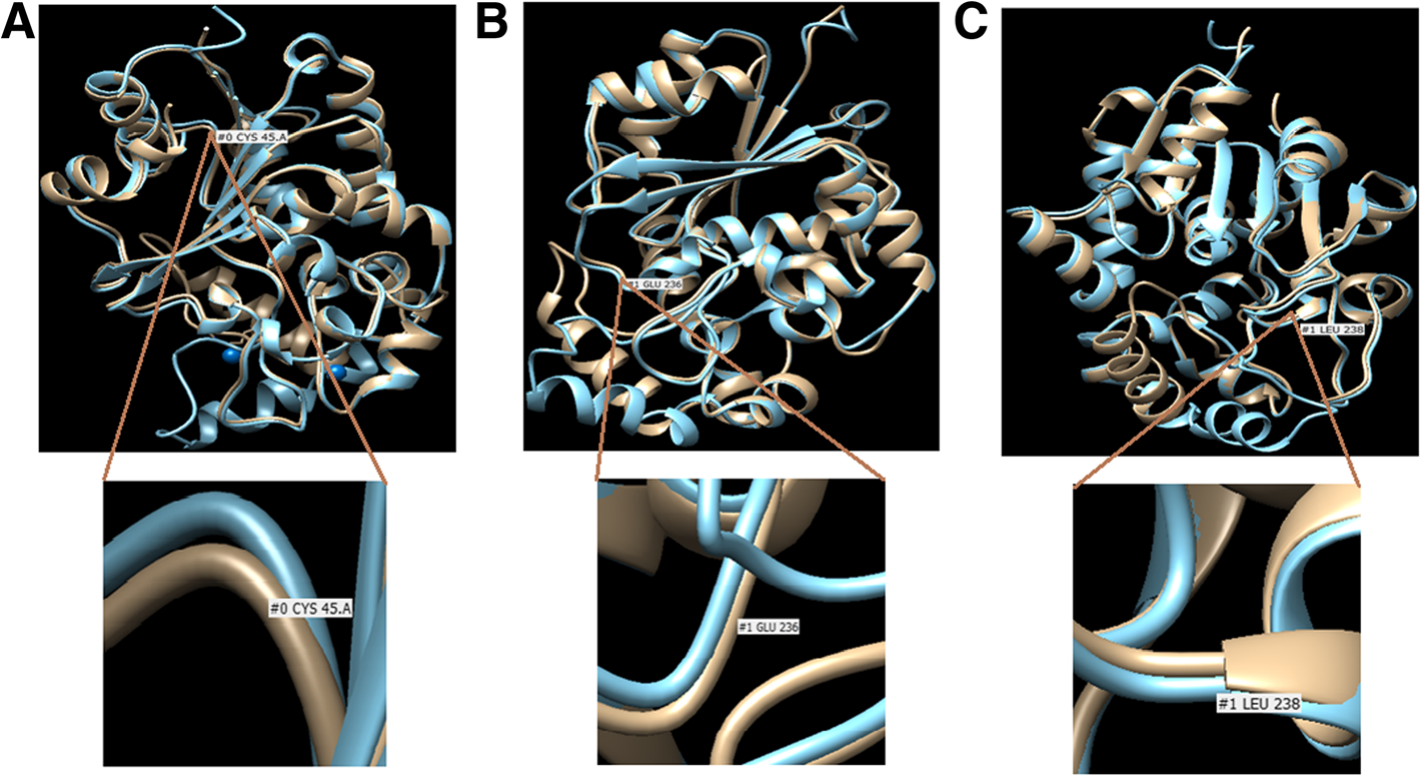
Homology modeling of novel missense variants identified in *PPT1* gene. The native structure (blue) and mutant structure (brown) are superimposed. **a** The model of p.Cys45Arg depicting the amino acid change from polar to basic at the codon number 45 (TGT-CGT). **b** The model of p.Val236Gly depicting the amino acid change from non-polar to acidic at the codon number 236 (GTG-GAG). **c** The model of p.Pro238Leu depicting the amino acide change from non-polar to hydrophobic at the codon number 238 (CCT-CTT). All the models reveal the conformational changes in the PPT1 protein structure.

The protein homology modeling of the missense point variants in the *PPT1* gene (p.Cys45Arg, p.Val236Glu, and p.Pro238Leu) and *TPP1* gene (p.Arg152Ser and p.Tyr459Ser) suggest their damaging effect at highly conserved residues. The variant p.Cys45Arg is in close proximity to Met41 and could affect the active site of PPT1 either by decreasing the oxyanion stabilization, altering the binding pocket or disrupting the active site by perturbing the position of Met41. The variant p.Val236Glu and p.Pro238Leu are very close to catalytic site Asp233. This could affect the N-linked glycosylation process and cause conformational changes in the protein (Fig. 3).

**Fig. 4:**
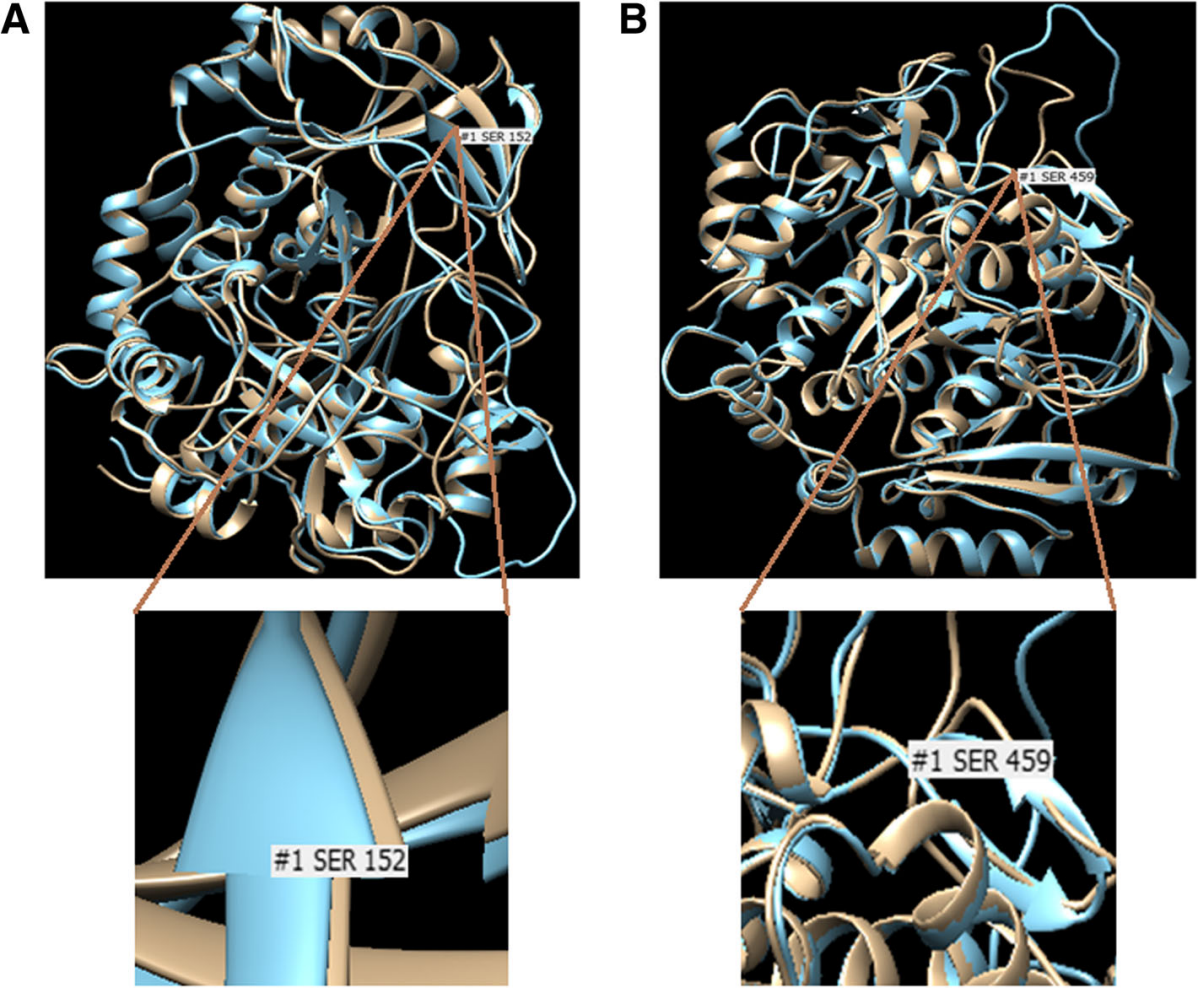
Homology modeling of novel missense variants identified in *TPP1* gene. The native structure (blue) and mutant structure (brown) are superimposed. **a** The model of p.Arg152Ser depicting the amino acid change from basic to polar at the codon number 152 (AGG-AGC). **b** The model of p.Tyr459Ser depicting the amino acid change from cyclic to non-cyclic at the codon number 459 (TAC-TCC). Both the models reveal the conformational changes in the TPP1 protein structure.

The amino acid substitution in the variant p.Arg152Ser causes disruption in β-strand conformation, which might disturb processing of TPP1. While the variant p.Tyr459Ser probably compromise the active center and destabilizes hydrophobic pocket (Fig. 4).

The novel variants of *PPT1* and *TPP1* genes were screened in 100 control individuals, however, none carried the given variants. The most common mutation p.Pro238Leu observed in 44% of patients with NCL1 and p.Arg206Cys observed in 26% of patients with NCL2 were screened in 100 unrelated healthy subjects and were found to have normal allele. The novel variants identified in the present study are submitted to the NCBI ClinVar repository [see Additional file 3].

## Discussion

Data presented here is the first study from India demonstrating the mutation spectrum of Batten disease (NCL1 and NCL2) in a large cohort. The given study reveals 34 cases of NCLs (12 with NCL1, and 22 with NCL2) with maximum NCL2 cases. Similarly, in a study by Santorelli et al., the highest numbers of cases confirmed the NCL2 (24%) [29].

The patients’ clinical appearance like seizures, myoclonic jerk, visual impairment, and neuroimaging examination showing cerebral atrophy and cerebellar atrophy were in concordance with the previously established phenotypes in NCL patients [3]. The mutations identified in this study resulted in a broader spectrum of clinical presentation and hence hampered the genotype-phenotype correlation in NCL patients. Such clinical presentations due to *PPT1* and *TPP1* gene mutations are also observed in the orthologous species. For instance, a study by Sanders et al. identified a homozygous mutation c.736_737insC in exon 8 of *PPT1* gene in a canine presenting NCL-like signs including, visual impairment, disorientation, behavioral changes, lack of PPT1 activity in the brain, and accumulation of autofluorescent lysosomal inclusions with the granular osmiophilic deposit in neurons [30]. Also, a study by Mahmood et al. established that a homozygous *TPP1* gene mutation in a zebrafish results in the progressive early onset of neurodegenerative phenotypes, small retina, accumulation of subunit c of mitochondrial ATP-synthase, and localized apoptotic cells death in the retina, optic tectum, and cerebellum [31].

Several country-specific mutations are reported in NCL1 and NCL2. The most common NCL1 mutation identified in Finland is p.Arg122Trp in *PPT1* gene, which accounts for 98% Finish variants [12]. A study by Das et al. revealed two common mutations, p.Arg151Ter and p.Thr75Pro, in *PPT1* gene of American NCL1 patients [32]. The absence of these common mutations in the present study of Indian patients suggests the molecular heterogeneity of NCL1 in India. In this study, a novel variant c.713C > T (p.Pro238Leu) was identified in the *PPT1* gene of four unrelated NCL1 positive families (44%) from the southern part of India. This suggests its possible founder effect in the Indian origin settlers. However, a detailed study in larger cohorts is essential.

In case of NCL2, two common mutations p.Arg208Ter and c.509-1G > C (as per old nomenclature T523-1G > C) accounting for approximately 60% of all identified *TPP1* mutant alleles worldwide and at least one of these mutation can be identified in more than 75% of patients [33, 34]. In the present study, one patient with NCL2 was identified with a homozygous p.Arg208Ter mutation but the variant c.509-1G > C was not observed in our cohort which indicate its uncommon occurrence in Indian NCL2 patients. The NCL2 country-specific mutation includes p.Gly284Val in Canada and p.Asp276Val in Argentina [14, 35]. In the present study, a known pathogenic mutation p.Arg206Cys was observed most commonly in the unrelated NCL2 patients (26%) suggesting its possible founder effect.

However, in the given study, the genetic diagnosis of about 17% of patients remained ambiguous. A similar percentage was also observed in previously published data were around 10% of patients were without any genetic identification [29]. This suggests that the deep intronic variants, large deletion or duplication in the NCL genes might also play a role in disease occurrence. In addition, as suggested by Santorelli et al., studying large informative families might identify new NCL genes and help in understanding NCLs molecular pathology [29].

A study by Das et al. established that a reduction in PPT1 and TPP1 enzyme activity ranges from 0 to 2.5% [33]. In the given study also, the reduction in these enzymes activity was from 0 to 2.8%. Based on these biochemical observations, the therapeutic approaches are tested to regain the enzyme activity. For instance, a study in a canine model with TPP1-deficiency revealed that the administration of a recombinant adeno-associated virus (rAAV) expressing canine TPP1 in the ependyma resulted in elevation of TPP1 expression leading to delay in clinical presentation and extension of life span [36]. In addition, studies to diminish the clinical phenotypes of NCL have been directed. Tracy et al. reported an alternative approach of using stem cell based delivery of therapeutic components to the retina, as the systemic administration would be ineffective [37]. This study reported the inhibition of the retinal degeneration in the canine model after a single intravitreal administration of autologous bone marrow-derived stem cells transduced with a TPP1 expression construct [37].

## Conclusions

The given study contributes four novel variants in *PPT1* gene and eight novel variants in *TPP1* gene mutation spectrum. Our results with remarkable heterogeneity provide new insight into the molecular pathology of NCL1 and NCL2. In addition, it was observed that the novel variant p.Pro238Leu was common in Indian NCL1 patients while a known pathogenic mutation p.Arg206Cys was commonly observed in Indian NCL2 patients. This can give a new insight into the molecular pathology of NCL patients with Indian origin.

## Additional files

Additional file 1: List of primers used for *PPT1 and TPP1* gene sequencing. The exons and the exon-intron boundaries of both the genes were bidirectionally sequenced using the given set of primers. (DOCX 13 kb)

Additional file 2: In silico analysis of the functional effect of the variants identified in the patients with NCL1 and NCL2. The in silico tools predicting the effect of DNA variants, coding non-synonymous variants, amino acid substitution, and non-coding variants were employed to predict the functional effect of the variants identified in the given study. (DOCX 17 kb)

Additional file 3: ClinVar Accession ID of the novel variants generated in the given study. The variants identified through Sanger sequencing are reported in NCBI ClinVar database. The file provides accession ID and the links to an individual variant. (DOCX 13 kb)

## Abbreviations

ExAc: Exome aggregation consortium
MLPA: Multiplex ligation-dependent probe amplification
NCBI: National Center for Biotechnology Information
NCL: Neuronal ceroid lipofuscinoses
PCR: Polymerase chain reaction
PPT1: Palmitoyl-protein thioesterase 1
TPP1: Tripeptidyl peptidase 1
