# Supplementary material for: Batten disease: biochemical and molecular characterization revealing novel PPT1 and TPP1 gene mutations in Indian patients
Source: BMC Neurol. 2018 Dec 12;18:203. doi: 10.1186/s12883-018-1206-1 (PMC6292089; doi:10.1186/s12883-018-1206-1)
Supplement: Supplementary file 1 — List of primers used for PPT1 and TPP1 gene sequencing. The exons and the exon-intron boundaries of both the genes were bidirectionally sequenced using the given set of primers. (DOCX 13 kb) [file 12883_2018_1206_MOESM1_ESM.docx]

**Primer sets for *PPT1* gene and *TPP1* gene Sanger sequencing**

| **Location** | **Primers** | | **Fragment Size (bp)** |
| --- | --- | --- | --- |
|  | **Sense 5'<---->3'** | **Antisense 3'<---->5'** |  |
| ***PPT1* gene** |  |  |  |
| Exon 1 | TGAAAGCTCCAGGGTAGGG | AGATGCGAACCCAGGCTAG | 337 |
| Exon 2 | GATAATGCTGTTTGAGGCCTC | CTGCTGCTGAAAACACAAGG | 289 |
| Exon 3 | TCAGTGGTTGTTTTCAGTCCC | TCCCTTCCAAGATAGGTGACA | 291 |
| Exon 4 | GTTTGGGGAGTCACAGAGGA | CTCCAGCAATGCTGGCTAGT | 330 |
| Exon 5 | TCTCACAGTGCCTTGTGCAT | ACGGTGACAGGTCTGTAATCT | 220 |
| Exon 6 | GACCTGTAGCTTGATCACCTCA | GAACGCACATCTATGGGAGC | 169 |
| Exon 7 | ATGGGGAAGAAACACAGTGG | TTTACTCTCCTGGCATGTGG | 250 |
| Exon 8 | TTGGCAGTATGTGCTGTGTG | TTCAGGAACTGGGAGCTGAA | 187 |
| Exon 9 | ACTCAGGACAAACTGCATTTTG | TTGCAAGCTGGATCTGAGCT | 336 |
| 3’-Promoter set 1 | CTTCCAAACCACATGGGAGA | CAACGTACTGAGAGAGGAAGGC | 473 |
| 3’-Promoter set 2 | GGTGATTTAACCAGTGCTTGG | CCTATTCTCTGCTAAAGCCAGC | 414 |
| 3’-Promoter set 3 | TTCCATTCTCGACCAACCTG | CAGAGTGGGGACTATGATTTCC | 365 |
| 3’-Promoter set 4 | TCCTTCTGGAGATCAACCCA | TGGCTGTACAGAAATGCAAA | 403 |
| ***TPP1* gene** |  |  |  |
| Exon 1 | GCAATCTTTCGAGGCAGATC | TCCCAATGTGTGCTCCCTCC | 451 |
| Exon 2 | GAGAGGGCTCGGGAGAAAGG | TTGGGGAGGATCCTAGGAGC | 368 |
| Exon 3 | CCGTGCCAGCTCCTAGTACG | GACATGATCGCCATCCCATG | 421 |
| Exon 4 | GAGGTCCAAAAGGGGGAGTT | CCTCTGAGCATCCCTGGGCA | 362 |
| Exon 5 | GATGGGAGGGAGTTGAGAGC | CTTATAGACTGTAATGCCCA | 389 |
| Exon 6 | TAGATGCCATTGGGGACTGG | GTCATGGAAATACTGCTCCA | 407 |
| Exon 7 | GCCTCCCCACAGTGTCCTCA | GGGATGGGCACAAAGATAGT | 411 |
| Exon 8 set 1 | ACTATCTTTGTGCCCATCCC | GTGTTGACCCGCTGGATGTA | 186 |
| Exon 8 set 2 | TGGCTCATGCTGCTCAGTAA | GGTCCCTACTGGAAGGTCTG | 281 |
| Exon 9 | CAGACCTTCCAGTAGGGACC | CTGTATCCCACACAAGAGAT | 396 |
| Exon 10 | AGTGTGCACAGTCACCTCGG | AACTTCGTTACAGCTTCCTC | 435 |
| Exon 11 | ATCCTCAGTTCAGCTGACTG | AGGTCAGGGGTTCTGAGTGA | 378 |
| Exon 12 | ACTTTAAAGCATCACTCCCA | AGGCTGGCATCAGATCTGGG | 370 |
| Exon 13 | GGGTGAGGAGATATACTCTT | GCATTTCAGGGTTAGGGAGA | 386 |
