# Supplementary material for: Batten disease: biochemical and molecular characterization revealing novel PPT1 and TPP1 gene mutations in Indian patients
Source: BMC Neurol. 2018 Dec 12;18:203. doi: 10.1186/s12883-018-1206-1 (PMC6292089; doi:10.1186/s12883-018-1206-1)
Supplement: Supplementary file 2 — In silico analysis of the functional effect of the variants identified in the patients with NCL1 and NCL2. The in silico tools predicting the effect of DNA variants, coding non-synonymous variants, amino acid substitution, and non-coding variants were employed to predict the functional effect of the variants identified in the given study. (DOCX 17 kb) [file 12883_2018_1206_MOESM2_ESM.docx]

***In silico* prediction of the functional effect of the variants identified in the patients with Batten disease (NCL1 and NCL2)**

| **Patient ID** | **cDNA position**  **(Amino Acid change)** | **MutationTaster2** | **SIFT** | **FATHMM** | **PolyPhen2** | **PROVEAN** | **Mutation Assessor^†^** |
| --- | --- | --- | --- | --- | --- | --- | --- |
| **P_1_** | Ex6:c.541G>A/p.V181M | Disease causing | Deleterious | Damaging | Probably damaging | Deleterious | High |
| **P_2_** | Ex3:c.329A>G/p.N110S | Disease causing | Deleterious | Damaging | Probably damaging | Deleterious | High |
| **P_3_** | Ex6:c.558G>A/p.W186* | Disease causing | NA | Pathogenic | NA | Deleterious | NA |
|  | Ex5:c.532_532delG/p.E178Nfs*13 | Disease causing | NA | NA | NA | NA | NA |
| **P_4_** | Ex7:c.713C>T/p.P238L | Disease causing | Deleterious | Damaging | Probably damaging | Deleterious | High |
| **P_5_** |  |  |  |  |  |  |  |
| **P_6_** |  |  |  |  |  |  |  |
| **P_7_** |  |  |  |  |  |  |  |
| **P_8_** | Ex2:c.133T>C/p.C45R | Disease causing | Deleterious | Damaging | Probably damaging | Deleterious | High |
| **P_9_** | Ex7:c.707T>A/p.V236G | Disease causing | Deleterious | Damaging | Probably damaging | Deleterious | High |
| **P_13_** | Ex8:c.1033A>C/p.M345L | Disease causing | Deleterious | Damaging | Probably damaging | Neutral | Low |
| **P_14_** | Ex8:c.1015C>T/p.R339W | Disease causing | Deleterious | Damaging | Probably damaging | Deleterious | High |
| **P_15_** | Ex8:c.1016G>A/p.R339Q | Disease causing | Deleterious | Damaging | Probably damaging | Deleterious | Medium |
| **P_16_** | Ex12:c.1546_1547delTT/p.F516* | Disease causing | NA | NA | NA | NA | NA |
| **P_17_** | Ex6:c.616C>T/p.R206C | Disease causing | Deleterious | Damaging | Probably damaging | Deleterious | High |
| **P_18_** |  | Disease causing |  |  |  |  |  |
| **P_19_** |  | Disease causing |  |  |  |  |  |
| **P_20_** |  | Disease causing |  |  |  |  |  |
| **P_21_** |  | Disease causing |  |  |  |  |  |
| **P_22_** | Ex6:c.622C>T/p.R208* | Disease causing | NA | Pathogenic | NA | Deleterious | NA |
| **P_23_** | Ex7:c.857A>G/p.N286S | Disease causing | Deleterious | Damaging | Probably damaging | Neutral | Medium |
|  | Ex3:c.184delT/p.S62Rfs*19 | Disease causing | NA | NA | NA | NA | NA |
| **P_24_** | Ex5:c.456G>C/p.R152S | Disease causing | Deleterious | Tolerated | Probably damaging | Deleterious | Medium |
| **P_25_** | Ex11:c.1376A>C/p.Y459S | Disease causing | Deleterious | Damaging | Probably damaging | Deleterious | High |
| **P_26_** |  |  |  |  |  |  |  |
| **P_27_** | Ex5:c.455_488del/p.S153Pfs*19 | Disease causing | NA | NA | NA | NA | NA |
| **P_28_** | Ex5:c.471C>A/p.Y157* | Disease causing | NA | Pathogenic | NA | Deleterious | NA |
| **P_29_** | Ex7:c.689_689delT/p.F230Sfs*28 | Disease causing | NA | NA | NA | NA | NA |
|  | Ex12:c.1449_1450insG/p.I484Dfs*7 | Disease causing | NA | NA | NA | NA | NA |
| **P_30_** | In4:g.2023_2024insT | Disease causing | NA | NA | NA | NA | NA |
| **P_31_** |  |  |  |  |  |  |  |

Abbreviations: The Functional Analysis Through Hidden Markov Models (FATHMM), Not Applicable (NA), Polymorphism Phenotyping version 2 (PolyPhen-2), Protein Variation Effect Analyzer (PROVEAN), The Sorting Intolerant from Tolerant (SIFT)

† Impact of amino acid substitution on protein function
