# Supplementary material for: Batten disease: biochemical and molecular characterization revealing novel PPT1 and TPP1 gene mutations in Indian patients
Source: BMC Neurol. 2018 Dec 12;18:203. doi: 10.1186/s12883-018-1206-1 (PMC6292089; doi:10.1186/s12883-018-1206-1)
Supplement: Supplementary file 3 — ClinVar Accession ID of the novel variants generated in the given study. The variants identified through Sanger sequencing are reported in NCBI ClinVar database. The file provides accession ID and the links to an individual variant. (DOCX 13 kb) [file 12883_2018_1206_MOESM3_ESM.docx]

The dataset generated and/or analyzed during the current study is available in the NCBI ClinVar repository. Following are the accession ID of the novel variants submitted to the ClinVar repository

1. **Variant c.532_532delG (p.Glu178Asnfs*13) in exon 5 of *PPT1* gene. (Identified in the patient P_3_)**

ClinVar Accession ID: SCV000282516.1

[<https://www.ncbi.nlm.nih.gov/clinvar/variation/236410/>]

1. **Variant c.713C>T (p.Pro238Leu) in exon 7 of *PPT1* gene. (Identified in the patients P_4_, P_5_, P_6_, and P_7_)**

ClinVar Accession ID: SCV000282513.1

[<https://www.ncbi.nlm.nih.gov/clinvar/variation/236407/>]

1. **Variant c.133T>C (p.Cys45Arg) in exon 2 of *PPT1* gene. (Identified in the patient P_8_)**

ClinVar Accession ID: SCV000282514.1

[<https://www.ncbi.nlm.nih.gov/clinvar/variation/236408/>]

1. **Variant c.707T>A (p.Val236Gly) in exon 7 of *PPT1* gene. (Identified in the patient P_9_)**

ClinVar Accession ID: SCV000282515.1

[<https://www.ncbi.nlm.nih.gov/clinvar/variation/236409/>]

1. **Variant c.184delT (p.Ser62Argfs*19) in exon 3 of *TPP1*gene. (Identified in the patient P_23_)**

ClinVar Accession ID: SCV000803215.1

[<https://www.ncbi.nlm.nih.gov/clinvar/variation/559497/>]

1. **Variant c.456G>C (p.Arg152Ser) in exon 5 of *TPP1* gene. (Identified in the patient P_24_)**

ClinVar Accession ID: SCV000262580.1

[<https://www.ncbi.nlm.nih.gov/clinvar/variation/221282/>]

1. **Variant c.1376A>C (p.Tyr459Ser) in exon 11 of *TPP1* gene. (Identified in the patient P_25_ and P_26_)**

ClinVar Accession ID: SCV000257502.2

[<https://www.ncbi.nlm.nih.gov/clinvar/variation/218336/>]

1. **Variant in c.471C>A (p.Tyr157Ter) exon 5 of *TPP1* gene. (Identified in the patient P_28_)**

ClinVar Accession ID: SCV000292001.1

[[https://www.ncbi.nlm.nih.gov/clinvar/variation/242357/](https://www.ncbi.nlm.nih.gov/clinvar/variation/242357/%20)]

1. **Variant in c.689_689delT (p.F230Sfs*28) exon 7 of *TPP1* gene. (Identified in the patient P_29_)**

ClinVar Accession ID: SCV000803213.1

[<https://www.ncbi.nlm.nih.gov/clinvar/variation/559477/>]

1. **Variant in c.1449_1450insG (p.Ile484Aspfs*7) exon 12 of *TPP1* gene. (Identified in the patient P_29_)**

ClinVar Accession ID: SCV000803212.1

[<https://www.ncbi.nlm.nih.gov/clinvar/variation/559478/>]
